# Supplementary material for: End Sequence Analysis Toolkit (ESAT) expands the extractable information from single-cell RNA-seq data
Source: Genome Res. 2016 Oct;26(10):1397–410. doi: 10.1101/gr.207902.116 (PMC5052061; doi:10.1101/gr.207902.116)
Supplement: Supplemental Material [file supp_gr.207902.116_Supp_Materials.tar.gz › ESAT-esat_v0.1/doc/html/classbroad_1_1pda_1_1samtools_1_1_wrapped_iterator.html]

ESAT: broad.pda.samtools.WrappedIterator Class Reference


ESAT


- Main Page
- Related Pages
- Packages
- Classes
- Files

- Class List
- Class Index
- Class Hierarchy
- Class Members

All Classes Namespaces Files Functions Variables Enumerator Pages

- broad
- pda
- samtools
- WrappedIterator

Public Member Functions |
List of all members

broad.pda.samtools.WrappedIterator Class Reference

Inheritance diagram for broad.pda.samtools.WrappedIterator:

[legend]

Collaboration diagram for broad.pda.samtools.WrappedIterator:

[legend]

|  |  |
| --- | --- |
| Public Member Functions | |
| void | close () |
|  | |
| boolean | hasNext () |
|  | |
| Alignment | next () |
|  | |
| void | remove () |
|  | |

## Detailed Description

Created by IntelliJ IDEA. User: jrobinso Date: Sep 22, 2009 Time: 2:32:51 PM To change this template use File | Settings | File Templates.

Copied to broad.pda because the IGV class is not visible outside of its own package

## Member Function Documentation

|  |  |  |  |  |
| --- | --- | --- | --- | --- |
| void broad.pda.samtools.WrappedIterator.close | ( |  | ) |  |

|  |  |  |  |  |
| --- | --- | --- | --- | --- |
| boolean broad.pda.samtools.WrappedIterator.hasNext | ( |  | ) |  |

|  |  |  |  |  |
| --- | --- | --- | --- | --- |
| Alignment broad.pda.samtools.WrappedIterator.next | ( |  | ) |  |

|  |  |  |  |  |
| --- | --- | --- | --- | --- |
| void broad.pda.samtools.WrappedIterator.remove | ( |  | ) |  |

---

The documentation for this class was generated from the following file:

- java/broad/pda/samtools/WrappedIterator.java


---

Generated on Fri Aug 15 2014 15:11:01 for ESAT by  

 1.8.7
